# Comparative Two-Year Outcomes of Three Different Three-Piece Intraocular Lenses in Flanged Intrascleral Fixation

**DOI:** 10.3390/medicina62101985

**Published:** 2026-10-14

**Authors:** Burhan Başkan, Yusuf Evcimen

**Affiliations:** Department of Ophthalmology, SBU Van Training and Research Hospital, 65300 Van, Turkey; yusufevcimen23@gmail.com

**Keywords:** Yamane technique, flanged intrascleral fixation, three-piece intraocular lens, corneal endothelial cell loss, IOL decentration, haptic angulation

## Abstract

*Background and Objectives*: To compare 2-year positional stability, endothelial preservation, and visual outcomes of three three-piece intraocular lenses (IOLs) implanted by the Yamane technique: Eyecryl (polypropylene haptics, 5° haptic angulation), Sensar OptiBlue AR40e (polymethyl methacrylate [PMMA] haptics, 5° haptic angulation), and AcrySof MA60AC (PMMA haptics, 10° haptic angulation). *Materials and Methods*: Records of 192 consecutive patients (one eye per patient; no bilateral implantation) receiving a study IOL were reviewed; 145 patients (75.5%) completed 24 months of follow-up (Eyecryl 48 eyes, Sensar 48 eyes, MA60AC 49 eyes). Endothelial cell density (ECD) loss and anterior segment OCT measures of decentration, tilt, and anatomical anterior chamber depth (ACD) were modelled with mixed-effects models weighted by stabilised inverse probability of treatment weights, using cluster-robust standard errors. *Results*: Visual acuity gain was equivalent within the prespecified ±0.15 logMAR margin (two one-sided tests, *p* = 0.009). Operative time differed (Eyecryl 12.9 ± 3.4 versus MA60AC 24.1 ± 7.6 min; *p* < 0.001), as did the proportion of eyes requiring repeat cautery of at least one haptic (Eyecryl 4.2%, Sensar 22.9%, MA60AC 42.9%; *p* < 0.001). MA60AC showed greater decentration (0.75 ± 0.21 versus 0.56 ± 0.20 mm) and shallower ACD (3.42 ± 0.38 versus 3.61 ± 0.35 mm); tilt did not differ (omnibus *p* = 0.312). Unadjusted 24-month ECD loss, expressed as a percentage of baseline ECD, was 4.92 ± 1.52% (Eyecryl), 6.18 ± 1.87% (Sensar), and 7.45 ± 2.01% (MA60AC) (all pairwise *p* < 0.0167). After operative duration, ACD, and decentration were entered, the MA60AC-versus-Eyecryl difference in ECD loss fell by 81.4%, from 2.1 to a non-significant 0.4 percentage points; this attenuation is descriptive, not causal mediation. *Conclusions*: All three platforms gave equivalent 2-year visual outcomes. Eyecryl showed better positional stability and lower unadjusted endothelial loss than MA60AC, with shorter surgery and a less anterior optic. Because haptic material and angulation vary together here, comparisons concern whole platforms, not individual attributes.

## 1. Introduction

Complete loss of posterior capsular and zonular support remains one of the most demanding situations in anterior segment surgery. Its causes are heterogeneous and include ocular trauma, hereditary lens subluxation associated with connective tissue disorders, iatrogenic capsular rupture during cataract surgery, and late spontaneous dislocation of the capsular complex. Although these aetiologies differ considerably in their presentation and in the age at which they occur, they converge upon a single technical problem: the intraocular lens (IOL) must be secured permanently and safely within an eye that lacks the anatomical anchor points around which most IOLs are designed [1,2].

Several salvage strategies are available, including anterior chamber IOLs, iris-fixated lenses, and various forms of scleral fixation. Among these, flanged intrascleral fixation has been adopted rapidly since Yamane and colleagues described the double-needle technique in 2017 [3]. In this procedure each haptic tip is externalised through a self-sealing scleral tunnel and converted into a heat-formed flange, which anchors the implant within the posterior chamber without sutures, without iris contact, and without the wound-related morbidity associated with angle-supported devices. Because the technique avoids suture erosion, knot exposure, and late suture breakage, it has become a valuable option for eyes that would otherwise be left aphakic [4,5,6].

Two design attributes of three-piece IOLs are likely to influence their behaviour within a scleral tunnel. The first is haptic material, together with the mechanical rigidity that derives from it. The technique was originally described using an implant with polyvinylidene fluoride (PVDF) haptics, a material that deforms plastically within the needle lumen and forms a smooth, reproducible terminal bulb when thermal cautery is applied [3,7]. Polymethyl methacrylate (PMMA) haptics are substantially stiffer, resist axial advancement through a 30-gauge lumen, and respond differently to cautery, tending to produce an irregular or charred terminus rather than a smooth flange [7,8]. These properties may prolong externalisation, necessitate repeated manipulation, and generate asymmetric radial forces within the scleral tunnel. The second attribute, which has received considerably less attention, is haptic angulation. Haptic angulation was designed for sulcus fixation: it displaces the optic posteriorly, away from the iris and towards the vitreous, to reduce iris contact and pupillary capture, so that a more angulated implant places its optic further behind the plane of haptic fixation [9]. How this geometry behaves once the haptics are externalised through scleral tunnels and anchored by flanges, rather than resting in the sulcus, has not been characterised. Any attribute that prolongs surgery or alters the final optic position may in turn affect long-term endothelial integrity, a consideration of particular importance because the corneal endothelium possesses no capacity for regeneration [10,11].

Three commercially available platforms are widely used in current practice and form the basis of the present analysis—the Eyecryl 3-Piece (Biotech Vision Care, Gujarat, India), the Sensar OptiBlue AR40e (Johnson & Johnson Vision, Santa Ana, CA, USA), and the AcrySof MA60AC (Alcon Laboratories, Fort Worth, TX, USA), the last of which is among the most frequently implanted three-piece IOLs worldwide [9]. According to the manufacturers’ technical documentation, the Eyecryl has flexible polypropylene haptics with approximately 5° haptic angulation, the Sensar has PMMA haptics with 5° angulation, and the MA60AC has PMMA haptics with 10° angulation; full specifications are given in the Methods. It should be noted that neither attribute is varied on its own across these three platforms. The two implants carrying rigid PMMA haptics (Sensar and MA60AC) share that material but differ in haptic angulation (5° vs. 10°), whereas the flexible-haptic Eyecryl differs from both in haptic material and manufacturer while sharing its 5° angulation with the Sensar. A three-group gradient can therefore not be assigned to a single design attribute. Whether differences in haptic architecture produce clinically meaningful divergence over a two-year period, and whether any such divergence is attributable to the haptic itself rather than to associated surgical factors such as operative time and lens position, has not previously been examined in a rigorous manner [12,13].

We therefore addressed this question in a consecutive cohort using propensity-score-weighted mixed-effects modelling. All three IOL types were implanted concurrently throughout the study period, which eliminates the chronological confounding that limits many retrospective IOL comparisons. We hypothesised that the three platforms would rank in the order Eyecryl, Sensar, MA60AC with respect to decentration, postoperative anterior chamber depth, and cumulative endothelial cell density (ECD) loss, following the anticipated order of intraoperative handling difficulty, from the flexible polypropylene haptic to the rigid PMMA haptics, with the more angulated PMMA platform expected to be the most demanding to externalise. We further examined whether any difference in ECD loss between platforms persisted after operative time and lens position had been entered into the model, since both variables plausibly lie between implant design and endothelial injury.

## 2. Materials and Methods

### 2.1. Study Design and Ethics

This was a retrospective analysis of prospectively maintained clinical records at a single tertiary referral centre (SBU Van Training and Research Hospital, Van, Turkey). The study was approved by the Van Training and Research Hospital Clinical Research Ethics Committee (approval number 2024/04-08; approval date 15 February 2024) and was conducted in accordance with the Declaration of Helsinki. The approval covered the retrospective review of records accrued from January 2022 onwards together with the subsequent scheduled follow-up, all of which formed part of routine clinical care. Written informed consent for surgical intervention was routinely obtained from all patients prior to operation. Because the study comprised a retrospective analysis of de-identified medical records, the ethics committee granted a formal waiver of individual informed consent for research participation. Reporting follows the STROBE guidance for observational studies; the completed STROBE checklist is provided as Supplementary Material Table S1.

### 2.2. Participants

Between January 2022 and January 2024, 238 consecutive patients presenting with complete absence of capsular and zonular support were assessed for eligibility. Forty-two were excluded before surgery: pre-existing corneal endothelial disease or guttata (*n* = 11), uncontrolled ocular hypertension or advanced glaucoma (*n* = 9), corneal opacification precluding reliable AS-OCT (*n* = 7), active or recurrent uveitis (*n* = 6), retained intraocular silicone oil (*n* = 5), and prior corneal surgery or keratoplasty (*n* = 4).

The remaining 196 patients (one eye each) were taken to surgery with the intention of implanting a study IOL by the Yamane flanged intrascleral technique, performed by a single surgeon. Four were converted intraoperatively to an alternative fixation strategy and were excluded in accordance with the prespecified criteria: one Sensar eye (haptic fracture during externalisation; converted to an iris-claw IOL) and three MA60AC eyes (two haptic fractures and one severe scleral tunnel tear during externalisation; all converted to sutured scleral fixation). A total of 192 patients therefore received the assigned study IOL and completed flanged intrascleral fixation (Eyecryl 64, Sensar 65, MA60AC 63).

All patients were scheduled for 24 months of postoperative follow-up as part of routine clinical care, with the last visit occurring no later than January 2026. After follow-up was complete, 145 patients (75.5% of those who received the assigned study IOL) had complete datasets and formed the primary analytical cohort (Eyecryl 48, Sensar 48, MA60AC 49); the flow of screening, allocation, attrition, and analysis is summarised in Figure 1. A prespecified sensitivity analysis using all 192 patients who received the assigned study IOL is reported in the Results.

Inclusion criteria: age ≥ 18 years; complete absence of capsular or zonular support confirmed intraoperatively; decision to proceed with the Yamane flanged intrascleral technique; and prospective records for best-corrected visual acuity (BCVA), manifest refraction, specular microscopy, and AS-OCT at baseline and at a minimum of one postoperative time point.

Exclusion criteria: pre-existing corneal endothelial disease (Fuchs endothelial dystrophy, posterior polymorphous dystrophy, or macular guttata); active or recurrent uveitis; uncontrolled ocular hypertension requiring more than three topical medications; prior keratoplasty; intraocular silicone oil; intraoperative conversion to an alternative fixation strategy; and significant corneal opacification precluding reliable AS-OCT imaging.

### 2.3. IOL Selection and Control of Confounding

IOL type was not randomised. In practice, selection was governed primarily by the availability of the required model and power in the hospital’s implant stock at the time of surgery; the three platforms were stocked concurrently and replenished without a fixed rotation. Clinical considerations played a secondary role: when more than one platform was available, the surgeon took into account the anticipated difficulty of haptic externalisation and the preoperative endothelial reserve, but no fixed selection algorithm was applied. Consistent with this, axial length, preoperative anterior chamber depth, the proportion of eyes with preoperative ECD below 1800 cells/mm^2^, and the rate of pars plana vitrectomy were similar across groups before weighting (Table 1; standardised mean differences 0.02–0.08). Although this pragmatic approach inherently introduces a risk of confounding by indication, the design was structured to minimise it. First, all three IOL types were implanted concurrently throughout the study period, with no interval during which a single type predominated, which pre-emptively reduced the period effect that might otherwise arise from sequential utilisation across different time windows. Second, the surgeon had completed more than 50 Yamane procedures prior to study initiation, limiting the influence of the learning curve on technical variability. Any remaining measurable baseline imbalance was addressed using inverse probability of treatment weighting (IPTW), as detailed below.

### 2.4. IOL Descriptions

Device specifications below are taken from the manufacturers’ technical data sheets and instructions for use.

Eyecryl 3-Piece (Biotech Vision Care, Gujarat, India): 6.0 mm hydrophobic acrylic optic, overall diameter 13.0 mm, flexible polypropylene modified-C haptics with approximately 5° anterior angulation.

Sensar OptiBlue AR40e (Johnson & Johnson Vision, Santa Ana, CA, USA): 6.0 mm hydrophobic acrylic optic with violet-light-filtering chromophore, overall diameter 13.0 mm, PMMA haptics with 5° anterior angulation.

AcrySof MA60AC (Alcon Laboratories, Fort Worth, TX, USA): 6.0 mm hydrophobic acrylic optic, overall diameter 13.0 mm, PMMA haptics with 10° anterior angulation.

All IOLs were implanted in their manufacturer-specified orientation; no intentional modification of angulation was performed. In all three implants the haptics are angled anteriorly relative to the optic, which places the optic posterior to the plane of the haptics; the angulation values above refer to this design feature.

### 2.5. Preoperative Assessment

Standardised preoperative evaluation comprised BCVA (Snellen converted to logMAR; counting fingers = 2.0, hand motion = 2.3, light perception = 2.7 logMAR); manifest refraction with spherical equivalent (SE = sphere + ½ × cylinder); Goldmann applanation tonometry; optical biometry (Lenstar LS 900, Haag-Streit AG, Köniz, Switzerland); non-contact specular microscopy (EM-4000, Tomey, Nagoya, Japan); AS-OCT (Solix FullRange OCT, Optovue/Visionix, Fremont, CA, USA); and dilated fundus examination. Optical biometry was used exclusively for axial length, keratometry, and anterior chamber depth in the context of IOL power calculation, and was not used for any assessment of IOL tilt or decentration.

### 2.6. IOL Power Calculation and Refractive Target

IOL power was calculated with the SRK/T formula using optical biometry (Lenstar LS 900). Because the plane of haptic fixation in the Yamane technique (tunnels 2.0 mm posterior to the limbus) lies anterior to the capsular bag, the manufacturer’s nominal in-the-bag A-constant was reduced by 0.7 for every platform, following the convention used for ciliary sulcus placement of three-piece IOLs. This uniform adjustment did not incorporate the posterior optic displacement produced by haptic angulation, which differs between the platforms (5° vs. 10°): Eyecryl 3-Piece 118.9 → 118.2; Sensar OptiBlue AR40e 118.7 → 118.0; AcrySof MA60AC 118.9 → 118.2. The intended postoperative target was mild myopia (−0.25 D to −0.50 D) in all eyes. The refractive prediction error was defined as the postoperative manifest spherical equivalent at 24 months minus the predicted spherical equivalent for the implanted power. The mean signed prediction error was used to identify any systematic myopic or hyperopic bias, and the mean absolute prediction error to describe predictability.

### 2.7. Surgical Technique

All operations were performed under peribulbar anaesthesia (2% lidocaine:0.5% bupivacaine 1:1, 8–10 mL) by a single vitreoretinal surgeon with more than 15 years of experience. Pars plana vitrectomy (PPV; 23- or 25-gauge; Constellation platform, Alcon, Fort Worth, TX, USA) or anterior vitrectomy was carried out when indicated by vitreous prolapse into the anterior chamber or the need for posterior segment visualisation.

Operative steps: a 2.8–3.2 mm clear corneal incision was created and the IOL inserted via an injector according to each manufacturer’s instructions. Two 30-gauge thin-wall needles (TSK Laboratory, Tochigi, Japan) were introduced 2.0 mm posterior to the limbus at opposing meridians, parallel to the iris plane, forming approximately 2–3 mm intrascleral tunnels. Each haptic was externalised using 25-gauge end-gripping forceps (DORC, Zuidland, The Netherlands). Flanges were buried within the scleral tunnels after symmetric centration of the optic. The corneal incision was hydrated and, where necessary, the conjunctiva closed with 8-0 polyglactin suture. Neither scleral flaps nor fixation sutures were used.

### 2.8. Flange Creation and Intraoperative Haptic Integrity

Because two of the three platforms studied (Sensar and MA60AC) carry PMMA haptics, and because PMMA does not form a flange as reproducibly as the PVDF haptic of the originally described technique, flange creation and haptic integrity were recorded explicitly.

A terminal bulb of approximately 0.3–0.4 mm diameter was created at each haptic tip with low-temperature disposable cautery (MST-210, Oertli, Berneck, Switzerland) applied for 1–2 s. Flange adequacy was assessed intraoperatively by the operating surgeon as a smooth, symmetric terminal bulb of sufficient diameter to resist retraction into the scleral tunnel on gentle traction. Where the first cautery application produced an irregular, charred, or undersized terminus, the manoeuvre was repeated; where the haptic fractured or shortened beyond the length required for tunnel seating, the haptic was re-externalised through a fresh tunnel where sufficient length remained; where it did not, the case was converted to an alternative fixation strategy and excluded from the study.

Three counts were recorded for every case and are reported by IOL group in the Results: the number of eyes in which at least one haptic required more than one cautery application; the number of intraoperative haptic fractures or flange failures in eyes that nonetheless retained the assigned implant; and the number of cases converted intraoperatively to an alternative fixation strategy and therefore excluded.

Operative time was recorded prospectively from first paracentesis or sclerotomy to final conjunctival closure, in minutes to the nearest half-minute, extracted directly from the electronic anaesthesia record. Because vitrectomy adds time independently of IOL type, operative times are reported separately for eyes undergoing PPV and eyes undergoing anterior vitrectomy, and vitrectomy modality was carried as a covariate in all multivariable models.

### 2.9. Postoperative Assessment

Patients were reviewed at postoperative day 1, week 1, and months 1, 3, 6, 12, 18, and 24. Each visit included BCVA, manifest refraction, Goldmann applanation tonometry, slit-lamp biomicroscopy, and dilated fundus examination. The measurement schedule for each longitudinal metric is specified below and was respected exactly in the mixed-effects models.

### 2.10. Anterior Segment OCT Analysis of IOL Position

IOL positional kinematics, comprising tilt (degrees), Euclidean decentration (mm), and anatomical anterior chamber depth (ACD, mm), were evaluated using high-resolution FullRange AS-OCT (Solix, Optovue/Visionix, Fremont, CA, USA). A single AS-OCT platform was used for all anterior segment imaging throughout the study; no other imaging device and no Purkinje-based instrument was used for positional analysis. Scans were acquired under standardised scotopic conditions (approximately 20 lux) without pharmacological mydriasis, in the horizontal (0–180°) and vertical (90–270°) meridians.

Tilt was defined as the angle between the IOL horizontal axis and the plane connecting the scleral spurs; anatomical ACD as the perpendicular distance from the corneal endothelial vertex to the anterior optic surface; and Euclidean decentration as the total vector distance between the IOL optic centre and the pupil vertex, averaged across three consecutive scans. In each meridian, the optic centre was defined as the midpoint of the line joining the two optic edges (the points at which the anterior and posterior optic surfaces meet), and the pupil vertex as the midpoint of the line joining the two pupillary margins. When only one optic edge was visible, the centre was assumed to lie 3.0 mm from the visible edge along the line connecting the two optic edges (i.e., the scan meridian), toward the presumed opposite edge. This corresponds to the radius of the manufacturer-specified 6.0 mm optic and assumes that the scan passed through the optic equator and that the full 6.0 mm diameter was represented in that meridian; measurements requiring this assumption were flagged. The horizontal (x) and vertical (y) offsets between the optic centre and the pupil vertex were measured on the 0–180° and 90–270° scans, respectively, and combined as √(x^2^ + y^2^). The pupil centre, rather than the visual axis, was used as the reference because the visual axis cannot be identified directly on AS-OCT images, whereas the pupillary margins are captured in the same scan as the optic edges; both landmarks could therefore be measured in a single image without registration to another device. Measurements were obtained at 3, 6, 12, 18, and 24 months (decentration and ACD) and at 6, 12, and 24 months (tilt). An additional unscheduled AS-OCT assessment was obtained whenever a patient reported symptoms suggestive of optic malposition before the next scheduled visit; measurements from unscheduled assessments were used only to inform the repositioning decision described below and were not entered into the longitudinal models, which used the scheduled time points exclusively.

Reference lines were placed manually on exported scans by two technicians working independently. Because the optic edge profile and haptic silhouette can betray implant identity on AS-OCT, scans were anonymised, cropped to remove the haptic silhouettes, and presented in randomised order for independent grading with an automated software overlay. The value entered into analysis was the arithmetic mean of the two graders’ measurements.

Inter-observer reliability was assessed in a random subset of 30 eyes using a two-way random-effects, absolute-agreement, single-measurement model (ICC_2,1_). The ICC was 0.71 (95% CI 0.58–0.82) for decentration and 0.91 (95% CI 0.84–0.95) for tilt. For decentration this corresponds to a standard error of measurement (SEM) of 0.11 mm and a minimum detectable change (MDC_95_ = 1.96 × √2 × SEM) of 0.30 mm for individual longitudinal tracking. The lower reliability of decentration relative to tilt reflects a recognised limitation of AS-OCT for this parameter, namely that the full 6 mm optic zone is not always visible within a single scan; decentration findings are therefore interpreted with corresponding caution throughout.

### 2.11. Endothelial Cell Density

Specular microscopy (EM-4000, Tomey) was performed at baseline, 12, and 24 months by technicians masked to IOL group assignment. Primary metrics were ECD (cells/mm^2^), percentage ECD loss = (preoperative − postoperative ECD)/preoperative ECD × 100, coefficient of variation in cell area (polymegathism), and proportion of hexagonal cells (hexagonality). At least three images per eye were acquired; automated cell-boundary detection was verified manually for ≥100 cells per scan.

Because postoperative specular microscopy was obtained only at 12 and 24 months, ECD-loss trajectories were modelled at these two post-baseline time points relative to the preoperative measurement. Percentage change from each eye’s own baseline was prespecified as the primary endothelial endpoint, because it removes between-patient variation in baseline cell density. In this cohort the within-patient correlation between baseline and 24-month absolute ECD was r = 0.981, which is what permits the paired percentage metric to have substantially lower variance than a cross-sectional comparison of absolute counts.

### 2.12. Repositioning Criteria

Surgical repositioning was considered only when Euclidean decentration was ≥0.8 mm on a scheduled or symptom-prompted unscheduled AS-OCT assessment and the patient reported attributable symptoms (monocular diplopia, disabling glare, or impaired contrast sensitivity). Because the distribution of decentration in the MA60AC group places an appreciable proportion of eyes above 0.8 mm, the symptom requirement, not the metric threshold alone, determined which eyes were operated. Asymptomatic patients with comparable decentration measurements were managed conservatively. This criterion was applied uniformly across all groups.

### 2.13. Statistical Analysis

Analyses were performed in R version 4.2.1 (R Foundation for Statistical Computing, Vienna, Austria) and SPSS version 28.0 (IBM Corp., Armonk, NY, USA). The Shapiro–Wilk test assessed distributional normality. Baseline comparisons used one-way ANOVA with Tukey HSD correction (normally distributed variables), the Kruskal–Wallis test with Dunn–Bonferroni correction (non-normal), and Fisher’s exact test (categorical, given expected cell counts < 5). Two-sided *p* < 0.05 was considered significant; for post hoc pairwise comparisons after ANOVA, Tukey HSD was used with a family-wise error rate of 0.05; for multiple secondary endpoints, Bonferroni correction was applied as appropriate, and uncorrected secondary comparisons are explicitly labelled as hypothesis-generating.

Missingness was evaluated using Little’s MCAR test. Sensitivity analyses employed pattern-mixture models with δ values of ±0.5% and ±1.0% for ECD loss to probe robustness under missing-not-at-random scenarios. Patients who developed new exclusion criteria during follow-up (*n* = 9) contributed their last available ECD values to the mixed model before censoring, treated as missing at random. A prespecified sensitivity analysis refitted the primary mixed model on all 192 patients who received the assigned study IOL, under the missing-at-random assumption.

Generalised propensity scores were estimated using a multinomial logistic regression model including age, sex, axial length, preoperative ECD, preoperative BCVA, surgical indication (aphakia, crystalline lens dislocation, IOL–capsular bag dislocation), vitrectomy modality, and baseline ACD. Stabilised weights were computed and truncated at the 1st and 99th percentiles, with a mean of 1.0 (range 0.72–1.34). Weighted balance was assessed using standardised mean differences (SMD), with SMD < 0.10 considered acceptable. Effective sample sizes after weighting, calculated as (Σw_i_)^2^/Σw_i_^2^, were 47.2 (Eyecryl), 46.8 (Sensar), and 48.1 (MA60AC).

Generalised propensity scores and stabilised weights were estimated with the WeightIt package (version 0.14.2). Weighted linear mixed-effects models were fitted with lme4::lmer (lme4 version 1.1-35.1), and all reported standard errors and confidence intervals for these models were obtained by cluster-robust CR2 (bias-reduced linearisation) estimation with Satterthwaite degrees of freedom using the clubSandwich package (version 0.5.11), clustering on the patient. This estimator is appropriate because inverse probability weighting induces heteroskedasticity, and because the residual variance of 24-month ECD loss differed across IOL groups (within-group SD 1.52% for Eyecryl, 1.87% for Sensar, and 2.01% for MA60AC). Weighted cross-sectional regressions were fitted as design-based models using survey::svydesign and survey::svyglm (survey version 4.4-2), which return Horvitz–Thompson (Huber–White) sandwich standard errors directly. Variance inflation factors (VIF), by contrast, are design-matrix diagnostics and are unaffected by the choice of standard-error estimator; they are reported alongside tolerance (1/VIF). Time was entered in years in all mixed models. For the weighted mixed model of 24-month BCVA change, the random-effects variance components were τ^2^_intercept_ = 0.0084, τ^2^_slope_ = 0.0031 (per year^2^), intercept–slope correlation ρ = −0.12, and σ^2^_residual_ = 0.0152.

BCVA equivalence was tested using two one-sided tests (TOST) with a prespecified ±0.15 logMAR margin. This margin corresponds to 1.5 lines on a logMAR chart and approximates the 95% test–retest limits of agreement reported for letter-chart visual acuity, so that a between-group difference inside it cannot be distinguished reliably from measurement variability [14,15]; because acuity in this cohort was measured on Snellen charts and converted to logMAR, a procedure with greater test–retest variability than ETDRS testing, the margin is not permissive for this dataset; equivalence required both one-sided tests to be significant at α = 0.05. The TOST was performed on the 24-month change in BCVA from baseline, using the IPTW-weighted linear contrast standard error from the mixed model (SE = 0.063). This model-based standard error is approximately twice the value that a naive two-sample calculation from the group standard deviations would yield, because it propagates the random-slope variance and the weighting structure; it is therefore the conservative choice, and equivalence declared under it would also hold under the smaller unweighted standard error. The variance components reported above, together with the design effect of the stabilised weights and the CR2 small-sample correction, account for this inflation.

A prespecified exploratory comparison of eyes with preoperative ECD < 1800 versus ≥1800 cells/mm^2^ is reported in the Supplementary Material only (Table S2). No correction for multiplicity was applied to this comparison or to any other secondary analysis, and with 16 eyes in the low-ECD stratum the family-wise type I error rate for the secondary endpoints as a set is substantially above 0.05. These analyses are therefore descriptive and are not used to support any conclusion.

An a priori sample size calculation was not performed; the cohort comprises all consecutive patients meeting the eligibility criteria, and the precision of the primary endpoint is conveyed by its 95% confidence interval (1.72–3.34).

### 2.14. Change in the IOL-Type Coefficient After Adjustment for Intermediate Variables

To describe how much of the ECD-loss difference between platforms was statistically accounted for by operative time, anatomical ACD, and decentration, we compared two IPTW-weighted multiple linear regression models for 24-month ECD loss.

- Model 1 (intermediate variables excluded): ECD loss ~ MA60AC + Sensar + age + axial length + preoperative ECD + PPV (yes/no).
- Model 2 (intermediate variables included): Model 1 covariates + operative time + anatomical ACD + decentration.

We report the change in the IOL-type coefficients between the two models as a descriptive quantity. This is not a causal mediation analysis: no indirect effect was estimated, no bootstrapped indirect-effect interval was computed, and the analysis carries none of the identification assumptions that such a decomposition would require. We therefore avoid the terms total effect, direct effect, and mediation throughout, and describe the finding as coefficient attenuation after the addition of intermediate variables. Model diagnostics included residual normality (Shapiro–Wilk), homoscedasticity (Breusch–Pagan), residual independence (Durbin–Watson), and VIF. All predictors met the prespecified VIF < 3.0 threshold except the MA60AC indicator in Model 2 (VIF 5.40); this is an expected consequence of entering an exposure indicator alongside variables that are strongly associated with it, and its interpretive consequences are addressed in the Discussion.

## 3. Results

### 3.1. Screening, Baseline Characteristics, Attrition, and Propensity Score Balance

Of the 238 patients assessed for eligibility, 42 were excluded before surgery and 196 were taken to theatre; 4 were converted intraoperatively and excluded, leaving 192 who received the assigned study IOL. Of these, 145 (75.5%) completed the 24-month visit. Attrition was balanced across groups (Eyecryl 16/64, 25.0%; Sensar 17/65, 26.2%; MA60AC 14/63, 22.2%; χ^2^ = 0.28, df = 2, *p* = 0.87). Completers and non-completers showed no significant differences in any baseline variable (all *p* > 0.10). Little’s MCAR test was non-significant (χ^2^ = 12.41, df = 13, *p* = 0.49), supporting the missing-at-random assumption. Pattern-mixture sensitivity analyses with δ = ±0.5% and ±1.0% yielded directionally identical results.

Reasons for postoperative attrition (*n* = 47) were geographic relocation or loss to contact (*n* = 22), voluntary withdrawal (*n* = 15), development of new exclusion criteria during follow-up (*n* = 9: Fuchs dystrophy de novo *n* = 3, uveitis relapse *n* = 4, retinal vein occlusion *n* = 2, evenly distributed across groups), and death from unrelated causes (*n* = 1). No patient withdrew because of dissatisfaction with the surgical outcome or an IOL-related complication. Attrition components are shown in Figure 1, with the cohort receiving the assigned study IOL as denominator (Eyecryl 64, Sensar 65, MA60AC 63).

Before IPTW, most covariates were already reasonably balanced (SMD < 0.20 for the majority). After applying stabilised weights truncated at the 1st–99th percentile distribution, all SMDs fell below 0.08, reflecting excellent weighted balance. The distribution of weights is provided in Supplementary Figure S1.

Baseline characteristics are presented in Table 1 with SMD values. Median age was 52.0 years (IQR 34.0–68.0), with a mean of 50.2 ± 22.8 years; the wide dispersion reflects an expected bimodal distribution comprising younger traumatic aphakia and older spontaneous dislocation. Male sex accounted for approximately 49%, mean axial length was 22.87 ± 1.26 mm, and mean preoperative ECD was 2058 ± 210 cells/mm^2^. The preoperative ECD distribution is shown in Supplementary Figure S2; 16 patients (11.0%) had values below 1800 cells/mm^2^. The dispersion of preoperative ECD in this cohort is narrower than is typical of unselected populations, which is attributable at least in part to the exclusion of eyes with pre-existing endothelial disease; the underlying per-eye values are available in the deposited dataset. Surgical indications (aphakia 53.8%; crystalline lens dislocation 16.6%; IOL–capsular bag dislocation 29.7%) and the rate of PPV (20.0%) were closely balanced across groups (all SMD < 0.04).

### 3.2. Visual and Refractive Outcomes

All three cohorts recovered vision robustly and to a statistically equivalent extent (Table 1; Figure 2A). Mean BCVA gain from baseline to 24 months was 0.30 ± 0.14 logMAR (Eyecryl), 0.31 ± 0.14 logMAR (Sensar), and 0.31 ± 0.19 logMAR (MA60AC; *p* = 0.860). TOST was applied to the IPTW-weighted, model-estimated MA60AC–Eyecryl contrast in 24-month BCVA change (weighted contrast 0.001 logMAR; SE = 0.063). Because this contrast is estimated from the weighted mixed model, it is not identical to the difference between the unweighted group means quoted above. The procedure yielded t_1_ = 2.41 (*p* = 0.009) and t_2_ = −2.38 (*p* = 0.009); the equivalence decision rests on the larger of the two *p*-values, 0.009, confirming that the difference fell within the ±0.15 logMAR margin. The Eyecryl–Sensar and Sensar–MA60AC comparisons were likewise within the equivalence margin (both *p* < 0.02). At 24 months, mean absolute BCVA was 0.22 ± 0.11 (Eyecryl), 0.24 ± 0.12 (Sensar), and 0.27 ± 0.14 logMAR (MA60AC; *p* = 0.314).

Refractive predictability was similar across groups but modest in absolute terms. The mean absolute SE prediction error at 24 months was 0.92 ± 0.70 D (Eyecryl), 1.00 ± 0.80 D (Sensar), and 1.15 ± 0.92 D (MA60AC; *p* = 0.594). The mean signed prediction error was +0.62 ± 0.98 D (Eyecryl), +0.68 ± 1.09 D (Sensar), and +0.79 ± 1.24 D (MA60AC; *p* = 0.728), indicating a predominantly hyperopic bias in all three groups; a myopic prediction error occurred in 11 of 48 (22.9%), 10 of 48 (20.8%), and 9 of 49 (18.4%) eyes, respectively (*p* = 0.842). Eyes within ±1.00 D of the refractive target numbered 27 of 48 (56.3%), 25 of 48 (52.1%), and 24 of 49 (49.0%) per group, respectively (*p* = 0.760); eyes within ±0.50 D numbered 18 of 48 (37.5%), 16 of 48 (33.3%), and 14 of 49 (28.6%), respectively (*p* = 0.638). The clinical implications of this level of predictability are addressed in the Discussion.

### 3.3. Operative Time

Operative times differed substantially and consistently across groups: Eyecryl 12.9 ± 3.4 min, Sensar 17.0 ± 6.7 min, MA60AC 24.1 ± 7.6 min (*p* < 0.001 for all pairwise comparisons). The same ordering was present within both vitrectomy strata (Table 2): among eyes undergoing anterior vitrectomy, 11.8 ± 2.6, 15.4 ± 5.1, and 22.3 ± 6.2 min (*p* < 0.001), and among eyes undergoing pars plana vitrectomy, 17.1 ± 3.1, 23.1 ± 6.8, and 32.1 ± 7.1 min (*p* < 0.001). The between-platform ordering is therefore not an artefact of differential vitrectomy use.

We consider haptic material the principal, but not the sole, contributor to these differences. The main source is likely the relative difficulty of externalising and flanging the PMMA haptics of the Sensar and MA60AC through 30-gauge needles: unlike a haptic that deforms plastically within the needle lumen, a stiff PMMA haptic resists axial force during externalisation and does not form a smooth terminal bulb as reliably on cautery, tending instead to require repeated application and additional manipulation to seat symmetrically within the scleral tunnel. This interpretation is supported directly by the intraoperative record: eyes in which at least one haptic required more than one cautery application numbered 2 of 48 (4.2%) for Eyecryl, 11 of 48 (22.9%) for Sensar, and 21 of 49 (42.9%) for MA60AC (*p* < 0.001), and intraoperative haptic fracture or flange failure occurred in 0 (0%), 1 (2.1%), and 4 (8.2%) retained eyes respectively (*p* = 0.087), with a further 1 Sensar and 3 MA60AC eyes converted intraoperatively and excluded. Haptic material alone cannot, however, account for the 7.1-min difference between the two PMMA platforms (Sensar and MA60AC), which share that material, and the higher rate of repeat cautery in MA60AC than in Sensar eyes (42.9% versus 22.9%) points to additional platform characteristics. With 10° angulation, the MA60AC haptic leaves the optic plane at a steeper angle relative to the needle path, which runs parallel to the iris plane, and may therefore require more manipulation to align the haptic tip with the needle lumen and to seat the flange symmetrically; differences in haptic configuration and tip geometry between manufacturers may also contribute. Because material and angulation co-vary across these platforms, their separate contributions cannot be quantified from the present data.

### 3.4. IOL Positional Kinematics

IPTW-weighted mixed models revealed significant group differences in decentration and anatomical ACD, but not in tilt (Table 3; Figure 2B,C,F).

At 24 months, MA60AC showed significantly greater decentration (0.75 ± 0.21 mm) than Eyecryl (0.56 ± 0.20 mm; mean difference 0.19 mm, 95% CI 0.07–0.31), a contrast that remained significant after Tukey adjustment for multiple comparisons. Sensar occupied an intermediate position (0.65 ± 0.19 mm) that did not differ significantly from either comparator after Bonferroni correction. Longitudinal tracking showed stable positioning for Eyecryl and Sensar from 3–6 months onwards, whereas MA60AC showed a small apparent drift, with decentration increasing by approximately 0.02 mm between 12 and 24 months (time × IOL type interaction versus Eyecryl, *p* = 0.04). This 0.02 mm change lies far below the MDC_95_ of 0.30 mm for individual tracking and should be regarded as within measurement noise. The 0.19 mm between-group difference also lies below the individual-level MDC_95_, and this apparent paradox deserves a plain statement. The MDC_95_ applies to a single measurement in a single eye; a group mean of roughly 48 eyes has a standard error smaller than that of one eye by a factor of √48, so a group-level difference of 0.19 mm is resolvable even though a 0.19 mm change in one patient would not be. Random measurement error of this kind is non-differential across groups; it inflates the standard error of the contrast and therefore reduces precision, but it does not bias the group mean difference in either direction, and the confidence interval reported here already carries that loss of precision. What follows is that these findings support statements about platforms and not about individual eyes: no clinician should conclude from this study that a given patient’s optic has moved.

MA60AC had the shallowest anterior chamber at 24 months (3.42 ± 0.38 mm) compared with Eyecryl (3.61 ± 0.35 mm; mean difference −0.19 mm, 95% CI −0.34 to −0.04; omnibus *p* = 0.02) and Sensar (3.52 ± 0.36 mm). This direction is opposite to that expected from haptic angulation alone, which displaces the optic posteriorly and would, other factors being equal, predict a deeper rather than a shallower anterior chamber for the 10°-angulated MA60AC (see Section 4).

Mean tilt at 24 months was 2.80 ± 1.65° (Eyecryl), 3.45 ± 2.10° (Sensar), and 3.85 ± 2.40° (MA60AC). The omnibus three-group effect was not significant (*p* = 0.312), and the 95% confidence interval for the MA60AC–Eyecryl contrast of 1.05° included zero (−0.18 to 2.28). The absence of a tilt difference despite greater translational and anterior displacement in the MA60AC group suggests that instability in the scleral tunnel manifests here primarily as centration offset and a shallower anterior chamber rather than angular misalignment, a mechanistically distinct mode of malposition with different implications for optical quality and for endothelial proximity.

The resulting group-mean geometry, namely a progressively shallower anterior chamber and a progressively more decentred optic from Eyecryl through Sensar to MA60AC, is illustrated to scale in Figure 3.

No significant intergroup difference was detected at 24 months (Eyecryl 0.68 ± 0.94 D; Sensar 0.85 ± 0.81 D; MA60AC 0.96 ± 0.92 D; *p* = 0.137).

### 3.5. Corneal Endothelial Cell Density

ECD loss diverged progressively across groups (Table 3; Figure 2D): 3.71 ± 1.32% (Eyecryl), 4.60 ± 1.58% (Sensar), 5.10 ± 1.89% (MA60AC). The omnibus three-group effect was significant (*p* < 0.001), but the pairwise structure was not uniform: Eyecryl differed from Sensar (*p* = 0.003) and from MA60AC (*p* < 0.001), whereas Sensar and MA60AC did not differ from each other at this time point (*p* = 0.160). The Eyecryl–MA60AC gap at 12 months was 1.39%.

Values were 4.92 ± 1.52% (Eyecryl), 6.18 ± 1.87% (Sensar), and 7.45 ± 2.01% (MA60AC). All three pairwise contrasts met the Bonferroni-corrected significance threshold of *p* < 0.0167; the two contrasts involving Eyecryl were significant at *p* < 0.001, whereas the Sensar–MA60AC contrast, the smallest of the three, cleared the corrected threshold by a narrower margin. The Eyecryl–MA60AC gap widened to 2.53% (95% CI 1.72–3.34; *p* < 0.001), indicating that divergence was progressive rather than confined to the early postoperative phase. The time × IOL type interaction was significant (*p* = 0.003).

Absolute 24-month values were 1952 ± 208 cells/mm^2^ (Eyecryl), 1940 ± 201 cells/mm^2^ (Sensar), and 1899 ± 202 cells/mm^2^ (MA60AC). The MA60AC–Eyecryl contrast was −53 cells/mm^2^ (95% CI −112 to +6; *p* = 0.082) (Figure 2E).

All groups maintained ECD well above the clinically accepted decompensation threshold of approximately 500 cells/mm^2^. The cross-sectional absolute difference between MA60AC and Eyecryl (−53 cells/mm^2^) did not reach significance, whereas the within-patient percentage loss difference (2.53%) was highly significant. The two metrics differ because percentage loss is a paired within-eye change referenced to each patient’s own baseline, and with a within-patient baseline–follow-up correlation of r = 0.981 it carries substantially lower variance than a cross-sectional between-group comparison of absolute counts against a pooled between-patient SD of approximately 200 cells/mm^2^. No patient developed persistent corneal oedema, bullous keratopathy, or required endothelial keratoplasty.

The MA60AC group exhibited greater polymegathism (coefficient of variation 33.8 ± 5.9% versus 29.1 ± 4.8% for Eyecryl; *p* = 0.003) and lower hexagonality (53.1 ± 7.1% versus 57.8 ± 6.2%; *p* = 0.014), both signs of endothelial stress. Sensar showed intermediate values that did not differ significantly from either comparator after Bonferroni correction.

### 3.6. Sensitivity Analysis in the Full Cohort Receiving the Assigned IOL

To address the possibility of attrition bias in the complete-case cohort, the primary mixed model was refitted on all 192 patients who received the assigned study IOL, under the missing-at-random assumption. The four intraoperatively converted patients are not included in this analysis; having never received a study IOL, they contributed no post-baseline observation and would in any case add nothing to the likelihood. Estimated marginal mean 24-month percentage ECD losses were 4.88% (Eyecryl), 6.12% (Sensar), and 7.39% (MA60AC; *p* < 0.001), and the regression coefficients for operative time, anatomical ACD, and decentration remained within 2% of their complete-case values. The complete-case analysis therefore does not appear to have introduced material bias.

### 3.7. Endothelial-Compromised Subgroup

A prespecified exploratory comparison in the 16 patients with preoperative ECD < 1800 cells/mm^2^ is not discussed further here. With approximately five to six eyes per group and no correction for multiplicity across this or the other secondary analyses, it does not support inference and is reported only for completeness.

### 3.8. Predictors of 24-Month ECD Loss and Coefficient Attenuation

Both IPTW-weighted multiple linear regression models fitted the data well. Model 2 (with intermediate variables) had R^2^ = 0.708, adjusted R^2^ = 0.688, F(9, 135) = 36.4, *p* < 0.001, with all diagnostic assumptions satisfied: residuals normally distributed (Shapiro–Wilk *p* = 0.26), homoscedastic (Breusch–Pagan *p* = 0.33), independent (Durbin–Watson 1.96), and variance inflation factors below the prespecified threshold of 3.0 for every predictor except the MA60AC indicator (VIF 5.40; tolerance 0.185), whose elevated value is an expected consequence of entering the exposure indicator together with variables strongly associated with it (Table 4).

In Model 1 (intermediate variables excluded), the MA60AC coefficient was 2.1 percentage points (95% CI 1.3 to 2.9; *p* < 0.001) and the Sensar coefficient was 1.2 percentage points (95% CI 0.5 to 1.9; *p* = 0.001), reproducing the hypothesised ordering across platforms (Model 1 fit: R^2^ = 0.524, adjusted R^2^ = 0.503, F(6, 138) = 25.3, *p* < 0.001). In Model 2 (operative time, anatomical ACD, and decentration added), the MA60AC coefficient fell to 0.4 percentage points (95% CI −0.1 to 0.9; *p* = 0.136), a relative attenuation of 81.4%. The Sensar coefficient attenuated in parallel, from 1.2 to 0.5 percentage points (95% CI −0.02 to 1.09; *p* = 0.057), a relative attenuation of 54.7%. Both IOL indicators therefore lost statistical significance once operative time and lens position were accounted for.

Three variables were associated with accelerated ECD loss in Model 2: operative duration (β = 0.042%/min, 95% CI 0.031–0.053; *p* < 0.001), shallower anatomical ACD (β = −1.280%/mm, 95% CI −1.639 to −0.921; *p* < 0.001), and greater decentration (β = 0.465%/mm, 95% CI 0.098–0.832; *p* = 0.014). PPV was independently associated with less ECD loss than anterior vitrectomy (β = −0.395%, 95% CI −0.625 to −0.165; *p* = 0.001), a finding discussed below. Preoperative ECD was also independently predictive in both models (Model 2 β = −0.0011% per cell/mm^2^, 95% CI −0.0021 to −0.0001; *p* = 0.041), indicating that eyes entering surgery with lower endothelial reserve sustained a proportionally greater loss; over a 500 cells/mm^2^ range this corresponds to a difference of approximately 0.55 percentage points. Age and axial length were not significant in either model.

### 3.9. Intraocular Pressure and Complications

IOP remained within the normal range throughout follow-up (24-month values: Eyecryl 14.2 ± 2.6, Sensar 14.5 ± 2.8, MA60AC 14.6 ± 2.9 mmHg; *p* = 0.841). No patient required escalation of glaucoma therapy or filtration surgery.

Intraoperative flange and haptic-integrity findings, postoperative complications, and attrition are summarised in Table 5. Postoperative complications were uncommon and did not differ significantly across groups. The composite complication endpoint occurred in 9 patients (18.8%) in the Eyecryl group, 11 (22.9%) in the Sensar group, and 15 (30.6%) in the MA60AC group (Fisher’s exact *p* = 0.422); because some patients experienced more than one event, the individual event counts in Table 5 exceed the number of affected patients. The numerically higher MA60AC rate was driven mainly by surgical repositioning (14.3% vs. 8.3% for Eyecryl; *p* = 0.726), undertaken only when decentration ≥ 0.8 mm was accompanied by patient-reported symptoms. All episodes of prolonged corneal oedema resolved with topical therapy; no eye progressed to chronic bullous keratopathy or required endothelial keratoplasty. No patient in any group developed endophthalmitis or complete IOL dislocation.

Repositioning was performed in 4 Eyecryl, 6 Sensar, and 7 MA60AC eyes, at a median of 4.5 months postoperatively (range 2–9 months). Repositioning therefore preceded the 6-, 12-, 18- and 24-month positional measurements in most cases. These eyes were retained in the mixed model, contributing their post-repositioning values at subsequent time points. As these eyes represented the most malpositioned cases, this approach is expected to attenuate rather than exaggerate the observed group differences in decentration. A prespecified sensitivity analysis excluding all repositioned eyes from all three groups showed a larger MA60AC–Eyecryl decentration difference of 0.26 mm (versus 0.19 mm in the primary analysis).

## 4. Discussion

In eyes without capsular support, flanged intrascleral fixation has now become a routine option [16]. However, the implant used is often determined by local availability rather than by design characteristics. The present cohort study clearly demonstrates that this choice is not a neutral one. Over the two-year follow-up period, the three platforms provided equivalent visual rehabilitation, and no corneal decompensation was observed in any case. Nevertheless, the platforms differed significantly with respect to operative time, optic centration, anterior chamber depth, and endothelial cell loss, and this differentiation followed the same ordinal pattern across all domains. Whether this ranking is directly attributable to the structural characteristics of the implants or is associated with secondary variables such as operative duration and final optic position remains an open question. Both operative time and final optic position varied according to the implant type, and it is considered that either factor may carry the signal for an adverse effect on the endothelium.

The positional behavior of the MA60AC platform differed significantly from that of the other two platforms. This divergence manifested as greater decentration and a shallower anterior chamber depth, as determined by direct measurements. The first plausible explanation for this finding relates to the haptic material. A rigid polymethyl methacrylate haptic resists the deformation that would permit a more compliant haptic to seat passively within the scleral tunnel. Furthermore, the asymmetrical terminal bulb of this haptic imposes an uneven load on the tunnel wall. Canabrava and colleagues arrived at a comparable conclusion through microstructural analysis of double-flanged haptics. The researchers demonstrated that flange geometry is largely dependent on the thermal behavior of the polymer [7].

This mechanism is specific to the flanged technique, because the originally described procedure employed a haptic selected in part for its thermoplastic behaviour under cautery, a property that polymethyl methacrylate does not share [17]. Our intraoperative record supports this interpretation, in that more than one cautery application was required in 42.9% of MA60AC eyes and 22.9% of Sensar eyes compared with 4.2% of Eyecryl eyes, and every intraoperative haptic fracture and every intraoperative conversion occurred in an eye receiving a polymethyl methacrylate haptic. Surgeons adopting these platforms should therefore anticipate repeated cautery application, should confirm flange adequacy before burying the haptic, and should have a salvage strategy available.

The shallower anterior chamber in the MA60AC group cannot be explained by its haptic angulation. Haptic angulation displaces the optic posteriorly, away from the iris and towards the vitreous, so that, with the haptics anchored at the same scleral location, a 10°-angulated implant would be expected to sit deeper than a 5°-angulated one. The observed direction is the opposite, which indicates that fixation-related factors outweighed the design geometry in this cohort. Plausible candidates are those already implicated in the decentration finding, namely less complete or asymmetric externalisation of a rigid haptic, which leaves a longer intraocular haptic segment, and irregular flanges that seat at different depths within the tunnel. These explanations are inferential, because intraocular haptic length and flange depth were not measured, and because no single design attribute is varied on its own across the three platforms, our data cannot attribute the finding to one attribute; separating them would require comparing implants matched on one attribute while differing in the other.

Tilt did not differ significantly between platforms, which indicates that the malposition associated with the MA60AC in this cohort was predominantly translational and anterior rather than angular. This distinction is clinically consequential, because tilt is a principal driver of coma and higher-order aberration whereas mild decentration is generally better tolerated optically, as Kimura and colleagues demonstrated using anterior segment tomography in pseudophakic eyes [18]. An anteriorly positioned optic, by contrast, lies closer to the corneal endothelium and thereby provides a plausible anatomical mechanism for the greater endothelial loss observed in that group.

In this study, operative time emerged as the variable demonstrating the most pronounced difference between the groups. MA60AC implantation required approximately twice as long as Eyecryl implantation. In the multivariate analysis, each additional minute of surgical time was associated with a 0.042 percentage-point increase in endothelial cell loss. Although per-minute effect coefficients are scarce in the scleral fixation literature, this finding is consistent with the results of Jo and colleagues, who demonstrated that endothelial damage is primarily dependent on the extent of intraocular manipulation [19]. On the other hand, in the retrospective study by Icoz and colleagues, which directly compared MA60AC with Eyecril Plus in 60 eyes, no significant difference was found between the groups with respect to operative time [20]. This discrepancy may be attributable to differences in sample characteristics and surgical conditions; however, we consider it necessary to address this discordance in the discussion. In that same study, although PVDF haptics were reported to offer potential advantages in terms of surgical ease of use, the overall efficacy and safety profile of MA60AC with PMMA haptics was reported to be similar. Taken together, we consider the longer operative time, and the additional intraocular manipulation it reflects, to be the most probable cause of the higher endothelial cell loss in the MA60AC group, rather than a direct effect of the PMMA haptic on the endothelium; haptic stiffness appears to act mainly through its effect on operative time. This interpretation agrees with the regression results described below, although, for the reasons given there, those results describe association rather than a formally identified causal pathway.

In our study, the endothelial loss rates observed at 24 months of 4.92%, 6.18%, and 7.45% were found to fall within the range of values reported in the literature for the flanged intrascleral fixation technique. In the original double-needle cohort, mean endothelial cell density declined from a preoperative value of 2341 cells/mm^2^ to 2240 cells/mm^2^ at 12 months and to 2189 cells/mm^2^ at 24 months, corresponding to cumulative losses of 4.3% and 6.5%, respectively [3]. The data from our present study encompass these reference values at both time points, with the Eyecryl platform exhibiting relatively lower loss rates and the MA60AC platform showing relatively higher loss rates compared with the aforementioned reference. As emphasized by LoBue and colleagues in their evaluation of flanged intrascleral haptic fixation, endothelial attrition following scleral fixation appears not to be confined to the early postoperative period but rather follows a cumulative course [21]. In keeping with this, the findings of our present study are consistent with this pattern. Indeed, the difference between the Eyecryl and MA60AC platforms increased from 1.39% at 12 months to 2.53% at 24 months. This observation demonstrates that the disparity in endothelial loss between the platforms becomes progressively more pronounced over time.

The coefficient for 24-month endothelial loss on the MA60AC platform was found to be 2.1 percentage points before the intermediate variables were entered into the model. After their addition, the coefficient decreased to 0.4 percentage points, representing an 81.4% attenuation. This finding suggests that the relationship between endothelial loss and the MA60AC is statistically accounted for largely by surgical and positional factors rather than by the implant material itself. The variables included in the model lie on the causal pathway between exposure and outcome, and the inclusion of such variables inherently attenuates the exposure coefficient. Perone and colleagues have noted the difficulty of distinguishing between over adjustment, collider bias, and unmeasured confounding in endothelial cell loss research [22]. The attenuation observed in our study is equally compatible with each of these alternative explanations.

A statistically nonsignificant adjusted coefficient does not imply the absence of an effect. The 95% confidence interval for the MA60AC coefficient extends from −0.1 to 0.9 percentage points, and its upper limit does not exclude an effect that could accumulate to a clinically meaningful degree over several decades. Furthermore, this analysis is not a causal mediation analysis, as we report only a change in the regression coefficient between two models, whereas causal mediation analysis requires an indirect effect with its own confidence interval. The contemporaneous measurement of the intermediate variables and the outcome also precludes a decomposition of the effect into variable specific pathways. Finally, the elevated variance inflation factor (VIF) for the MA60AC indicator in Model 2 indicates that the IOL-type indicator and the intermediate variables carry substantially overlapping information. Consequently, these coefficients should be interpreted as a set rather than as separately identified contributions. The VIF values for the three intermediate variables are 2.26, 1.93, and 1.44, respectively; these values do not indicate instability. However, the partitioning of variance between IOL type and these variables is poorly identified. The Sensar coefficient similarly decreased from 1.2 to 0.5 percentage points, and the adjusted coefficients for the two PMMA platforms almost entirely overlap. In this regard, the difference between the two platforms does not appear interpretable as evidence of distinct mechanisms.

All three platforms demonstrated similarly limited performance with respect to refractive predictability. The mean absolute prediction error approached or exceeded 1.00 D, and approximately half of the eyes achieved a final refraction within ±1.00 D of the target. This finding is consistent with the well-recognized difficulty of predicting effective lens position in eyes without capsular support. In such eyes, the axial optic position is determined by tunnel location, haptic angulation, and flange seating, rather than by capsular contraction. Hayashi and colleagues documented a similar relationship between lens position and refractive error in transscleral sutured fixation and reported comparable variability [12]. Although a measurable difference of 0.19 mm in anatomical anterior chamber depth was observed in our study, no significant between-group difference was found in refractive outcomes. This finding suggests that, beyond the hyperopic bias shared by all three platforms and attributable to the A-constant adjustment, the remaining prediction error is driven mainly by patient-level factors rather than by platform-level variability.

Patients should be counselled preoperatively that a residual refractive error of approximately one dioptre is common and that spectacle correction, or an additional refractive procedure in selected cases, may be required. The uniform 0.7 reduction applied to each manufacturer’s nominal A-constant followed the convention for sulcus placement, which assumes an optic lying anterior to the capsular bag. It did not account for the posterior optic displacement produced by haptic angulation, which moves the optic away from the iris and towards the vitreous. The mean signed prediction errors (+0.62, +0.68 and +0.79 D) show a predominantly hyperopic bias in all three groups, with 77–82% of eyes falling on the hyperopic side of the prediction, indicating that the effective lens position lay posterior to the position assumed by this convention and that the A-constant should have been increased rather than reduced. The numerically largest shift in the MA60AC group is in the direction expected from its greater angulation, although the between-group difference was not significant (*p* = 0.728) and the measured anatomical ACD was shallower, not deeper, in that group. In retrospect, the reduction was not appropriate, and constants should be derived from measured outcomes for each platform rather than from design assumptions. Since tunnel position was standardised, the residual error is largely attributable to the effective lens position. The constants can therefore be optimised by means of back-calculation. This method aims to compute, for each platform, the A-constant that drives the mean prediction error to zero while also minimising the mean absolute error. With only 48 to 49 eyes per platform, these constants would be provisional in nature, as approximately 100 eyes are generally required for stable estimation. Nevertheless, these constants would be specific to both the implant platform and the surgical technique, whereas manufacturer provided constants do not offer this level of specificity.

Findings regarding the endothelial protective effect of pars plana vitrectomy should be interpreted with caution. The most likely proposed mechanism for this relationship is that residual vitreous strands in eyes undergoing anterior vitrectomy may facilitate the passage of inflammatory mediators, thereby sustaining low-grade anterior segment inflammation. An alternative explanation pertains to hydrodynamic effects: the irrigation fluid used during anterior vitrectomy may create a flow profile closer to the endothelial surface, leading to higher shear stress for equivalent fluid volumes. However, neither potential mechanism has been confirmed yet. Furthermore, patients undergoing vitrectomy were selected based on clinical rationale. Consequently, the current data do not provide evidence supporting prophylactic pars plana vitrectomy to prevent endothelial damage [23,24].

This study has several limitations. The treatments administered to the patients were not randomized. Statistical methods control only for measurable variables; therefore, the results reflect statistical associations rather than direct effects attributable to implant selection. Haptic material and haptic angulation, and hence the degree of posterior optic vaulting, vary together across the three platforms, making it impossible to evaluate the isolated effect of any single design feature. Decentration was measured with an inter-observer agreement coefficient of 0.71. Although group means remain comparable at this level of agreement, longitudinal tracking of individual eyes is not feasible. The statistical correction for decentration is also incomplete, introducing measurement error into the 81.4% attenuation observed in the MA60AC coefficient. Apart from surgical duration, intermediate variables and outcomes were measured at the same visit, precluding the establishment of a temporal sequence among variables. Surgical repositioning due to decentration was performed in 4 eyes in the Eyecryl group, 6 eyes in the Sensar group, and 7 eyes in the MA60AC group; this procedure was accounted for across all statistical models. The attrition rate was 24.5%. Specular microscopy measurements were obtained only at baseline, month 12, and month 24. The A-constant was reduced uniformly across platforms, which produced a predominantly hyperopic prediction error, and the adjustment did not account for their different haptic angulations. Decentration was referenced to the pupil centre, which does not coincide with the visual axis and may shift with pupil diameter; the values therefore describe the position of the optic relative to the pupil rather than optical decentration relative to the line of sight. Secondary endpoints were analyzed without adjustment for multiple comparisons, and functional visual quality was not assessed. Finally, this was a single-surgeon, single-center study with a follow-up limited to 24 months. Long-term complications such as flange erosion, late haptic fracture, and polyolefin degradation fall outside this timeframe, highlighting the need for multicenter validation studies.

## 5. Conclusions

At the 24-month follow-up, the Eyecryl platform demonstrated superior positional stability, less decentration, and lower crude endothelial cell loss compared to the MA60AC. Nevertheless, all three implants yielded equivalent visual outcomes. These differences were accompanied by longer surgical duration and a more anterior optical location. Although a completely independent platform effect cannot be ruled out, this difference appears to largely co-occur with these surgical and positional factors. Because haptic material and haptic angulation (posterior optic vaulting) co-vary across the three evaluated platforms, the comparisons in this study pertain to the platforms as a whole rather than to individual design features. The practical implication is that optimizing surgical workflow in rigid-haptic implants (reducing duration, controlling flanges, ensuring optical centration) may be as decisive for corneal preservation as implant selection itself. Refractive predictability remains limited across all platforms, underscoring the necessity of preoperative patient counseling and of platform-specific A-constants. To validate these findings and disentangle the effects of haptic angulation from those of haptic material, randomized controlled trials comparing implants matched for angulation but differing in haptic material are required.

## Figures and Tables

**Figure 1:**
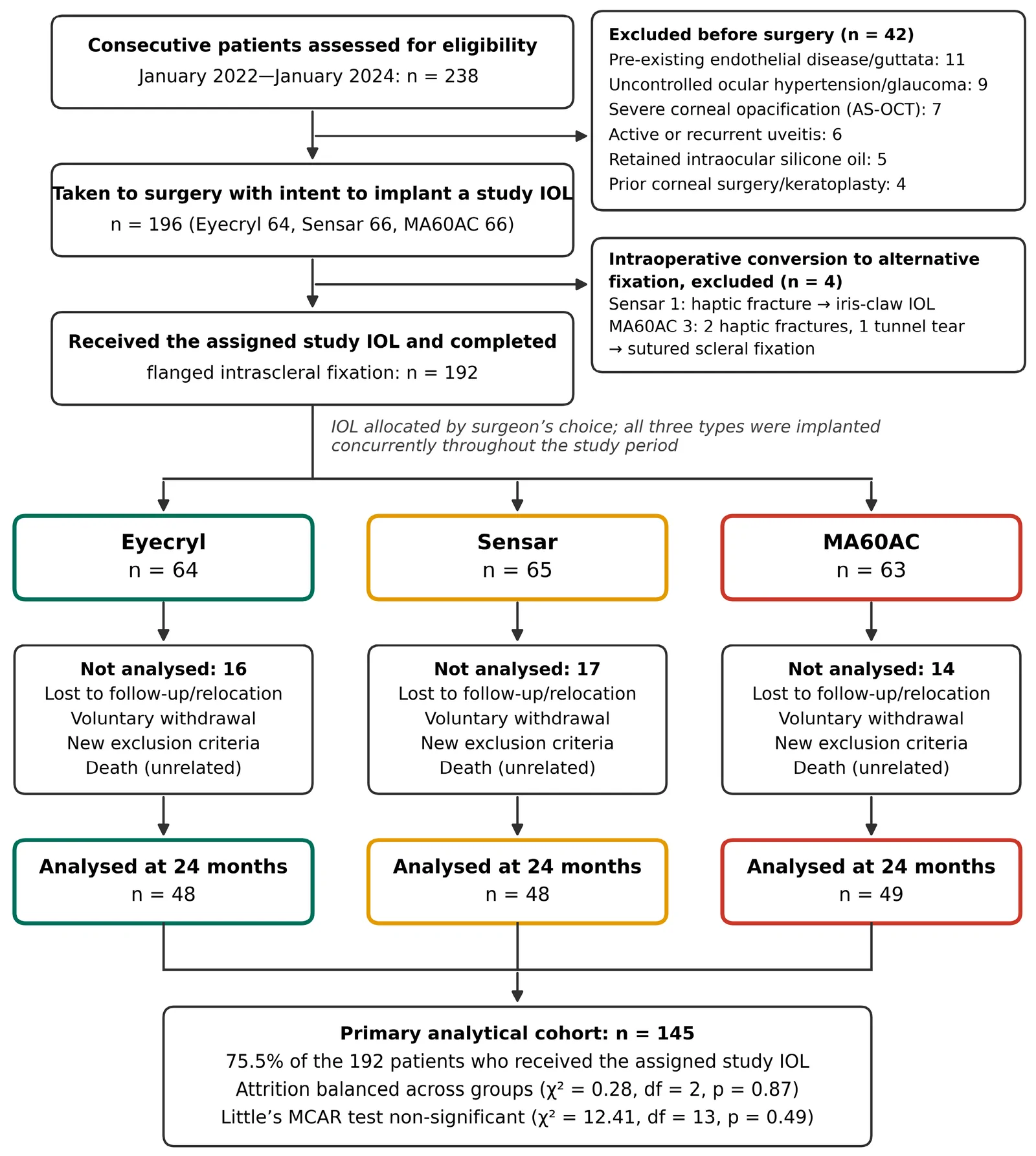
Patient flow diagram according to the STROBE guidelines. Postoperative attrition components across all groups (*n* = 47): relocation or loss to contact 22, voluntary withdrawal 15, new exclusion criteria 9 (de novo Fuchs dystrophy 3, uveitis relapse 4, retinal vein occlusion 2), death from unrelated causes 1.

**Figure 2:**
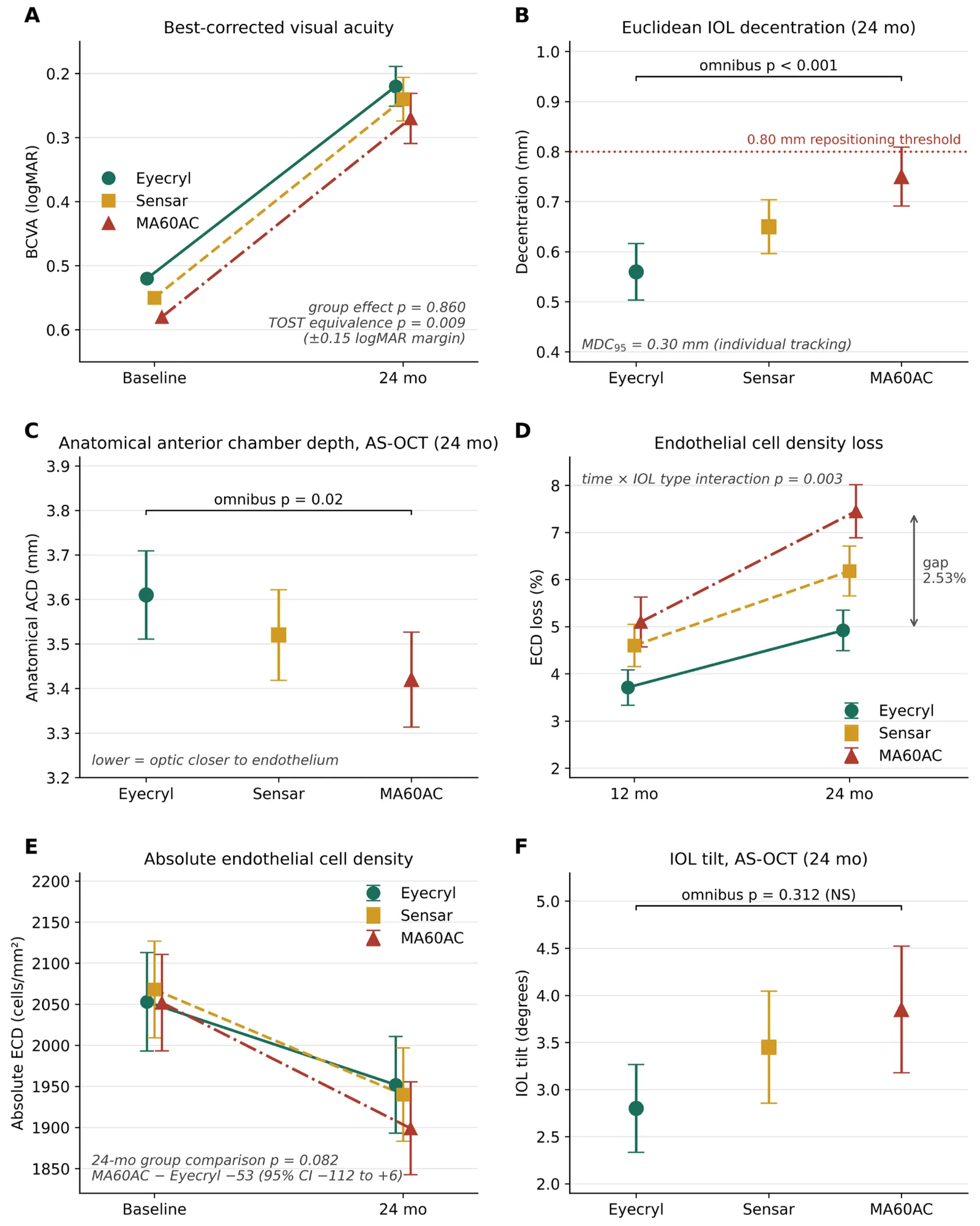
IPTW-weighted clinical outcomes over 24 months. (**A**) Best-corrected visual acuity at baseline and 24 months; (**B**) Euclidean IOL decentration at 24 months; (**C**) anatomical anterior chamber depth on AS-OCT at 24 months; (**D**) endothelial cell density loss at 12 and 24 months; (**E**) absolute endothelial cell density at baseline and 24 months; (**F**) IOL tilt on AS-OCT at 24 months. Markers denote group means; error bars denote 95% confidence intervals of the mean. The vertical axis of panel (**E**) is truncated for legibility; every eye in every group remained far above the ~500 cells/mm^2^ decompensation threshold. Baseline BCVA in panel (**A**) is plotted as the group mean without an interval, as the baseline dispersion is reported in Table 1. Lines connect the baseline and 24-month IPTW-weighted means of each group (solid: Eyecryl; dashed: Sensar; dash-dotted: MA60AC); error bars indicate 95% confidence intervals.

**Figure 3:**
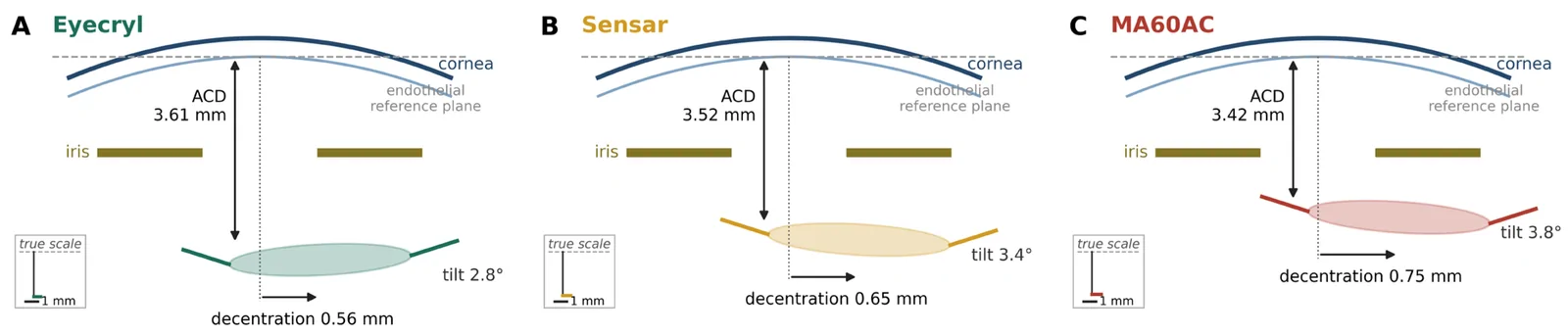
Schematic of IOL position at 24 months. (**A**) Eyecryl; (**B**) Sensar; (**C**) MA60AC. Main panels are schematic: between-group differences in anterior chamber depth and decentration are exaggerated (×6 and ×2.6) for visibility. The inset in each panel reproduces the same geometry to true scale against a 1 mm bar. Anterior chamber depth is measured from the corneal endothelial reference plane to the anterior optic surface. The iris is placed at an identical illustrative depth in all panels and is not a measured value. Tilt is drawn at its true angle; the three-group difference in tilt was not statistically significant (*p* = 0.312). These are schematic drawings constructed from group mean values, not patient-specific image captures.

**Table 1:** Baseline demographic, biometric and clinical characteristics by intraocular lens platform.

Parameter	Eyecryl (*n* = 48)	Sensar AR40e (*n* = 48)	AcrySof MA60AC (*n* = 49)	*p*	SMD Pre	SMD Post
*Demographic characteristics and ocular biometry*						
Age, years, mean ± SD	50.1 ± 22.5	50.3 ± 23.1	50.2 ± 22.9	0.999	0.02	0.01
Sex, male, *n* (%)	24 (50.0)	23 (47.9)	24 (49.0)	1.000	0.04	0.01
Axial length, mm	22.85 ± 1.24	22.88 ± 1.28	22.88 ± 1.27	0.991	0.02	0.01
Preoperative anterior chamber depth, mm	3.12 ± 0.32	3.15 ± 0.34	3.10 ± 0.31	0.746	0.08	0.02
*Corneal endothelium and visual function at baseline*						
Preoperative ECD, cells/mm^2^	2053 ± 212	2068 ± 208	2052 ± 210	0.916	0.076	0.01
Preoperative ECD below 1800 cells/mm^2^, *n* (%)	5 (10.4)	5 (10.4)	6 (12.2)	1.000	0.05	0.02
Preoperative BCVA, logMAR	0.52 ± 0.18	0.55 ± 0.19	0.58 ± 0.21	0.316	0.12	0.03
*Surgical indication*						
Aphakia (traumatic or iatrogenic), *n* (%)	26 (54.2)	26 (54.2)	26 (53.1)	1.000	0.03	0.01
Crystalline lens subluxation, *n* (%)	8 (16.7)	8 (16.7)	8 (16.3)	1.000	0.01	0.01
IOL and capsular bag complex dislocation, *n* (%)	14 (29.2)	14 (29.2)	15 (30.6)	1.000	0.03	0.01
*Vitrectomy modality*						
Anterior vitrectomy, *n* (%)	38 (79.2)	38 (79.2)	40 (81.6)	0.933	0.05	0.02
Pars plana vitrectomy, *n* (%)	10 (20.8)	10 (20.8)	9 (18.4)	0.933	0.05	0.02

Values are mean ± SD or *n* (%). Continuous variables were compared by one-way ANOVA and categorical variables by Fisher’s exact test with the Freeman–Halton extension. Rows with identical counts in all three groups return an exact *p* of 1.000 by construction. SMD pre and SMD post denote the largest pairwise standardised mean difference before and after inverse probability of treatment weighting. Balance was judged on SMD rather than on significance testing. Effective sample size after weighting was 47.2, 46.8 and 48.1, respectively. Haptic material and anterior angulation were Eyecryl polypropylene 5°, Sensar AR40e PMMA 5°, and MA60AC PMMA 10°. Median age for the whole cohort was 52.0 years (interquartile range 34.0 to 68.0). ANOVA, analysis of variance; BCVA, best-corrected visual acuity; ECD, endothelial cell density; IOL, intraocular lens; PMMA, polymethyl methacrylate; SD, standard deviation; SMD, standardised mean difference.

**Table 2:** Operative time stratified by vitrectomy modality.

Operative Time, min, by Subgroup	Eyecryl	Sensar AR40e	AcrySof MA60AC	*p*
Anterior vitrectomy (*n* = 38/38/40)	11.8 ± 2.6	15.4 ± 5.1	22.3 ± 6.2	<0.001
Pars plana vitrectomy (*n* = 10/10/9)	17.1 ± 3.1	23.1 ± 6.8	32.1 ± 7.1	<0.001
All eyes (*n* = 48/48/49)	12.9 ± 3.4	17.0 ± 6.7	24.1 ± 7.6	<0.001

Values are mean ± SD in minutes. Groups were compared by one-way ANOVA; the *p* column reports the omnibus three-group effect. All three pairwise contrasts were significant at *p* < 0.001 within each subgroup and overall (Tukey HSD correction). Stratum-weighted means reproduce the pooled means exactly: 12.90, 17.00 and 24.10 min respectively. ANOVA, analysis of variance; HSD, honestly significant difference; SD, standard deviation.

**Table 3:** Visual, refractive, positional and endothelial outcomes at 24 months.

Parameter at 24 Months	Eyecryl (*n* = 48)	Sensar AR40e (*n* = 48)	AcrySof MA60AC (*n* = 49)	MA60AC Minus Eyecryl (95% CI)	*p*
*Visual and refractive outcomes*					
BCVA gain from baseline, logMAR	0.30 ± 0.14	0.31 ± 0.14	0.31 ± 0.19	0.001 (−0.122 to 0.124)	0.860
Absolute BCVA, logMAR	0.22 ± 0.11	0.24 ± 0.12	0.27 ± 0.14	0.05 (−0.01 to 0.11)	0.314
Mean absolute spherical equivalent prediction error, D	0.92 ± 0.70	1.00 ± 0.80	1.15 ± 0.92	0.23 (−0.10 to 0.56)	0.594
Eyes within ±1.00 D of refractive target, *n* (%)	27 (56.3)	25 (52.1)	24 (49.0)	−7.3 (−27.2 to 12.6)	0.798
Eyes within ±0.50 D of refractive target, *n* (%)	18 (37.5)	16 (33.3)	14 (28.6)	−8.9 (−27.4 to 9.5)	0.649
Surgically induced astigmatism, D	0.68 ± 0.94	0.85 ± 0.81	0.96 ± 0.92	0.28 (−0.12 to 0.68)	0.137
*IOL positional kinematics on anterior segment OCT*					
Euclidean decentration, mm	0.56 ± 0.20	0.65 ± 0.19	0.75 ± 0.21	0.19 (0.07 to 0.31)	0.002
Anatomical anterior chamber depth, mm	3.61 ± 0.35	3.52 ± 0.36	3.42 ± 0.38	−0.19 (−0.34 to −0.04)	0.020
IOL tilt, degrees	2.80 ± 1.65	3.45 ± 2.10	3.85 ± 2.40	1.05 (−0.18 to 2.28)	0.312
*Corneal endothelial outcomes*					
ECD loss at 12 months, %	3.71 ± 1.32	4.60 ± 1.58	5.10 ± 1.89	1.39 (0.74 to 2.04)	<0.001
ECD loss at 24 months, % (primary endpoint)	4.92 ± 1.52	6.18 ± 1.87	7.45 ± 2.01	2.53 (1.72 to 3.34)	<0.001
Absolute ECD, cells/mm^2^	1952 ± 208	1940 ± 201	1899 ± 202	−53 (−112 to 6)	0.082 *
Polymegathism (coefficient of variation in cell area), %	29.1 ± 4.8	31.2 ± 5.2	33.8 ± 5.9	4.7 (2.1 to 7.3)	0.003 *
Hexagonality, %	57.8 ± 6.2	55.4 ± 6.8	53.1 ± 7.1	−4.7 (−7.8 to −1.6)	0.014 *
*Intraocular pressure*					
Intraocular pressure, mmHg	14.2 ± 2.6	14.5 ± 2.8	14.6 ± 2.9	0.4 (−0.7 to 1.5)	0.841

Values are mean ± SD or *n* (%). Continuous outcomes were modelled with IPTW-weighted linear mixed-effects models using cluster-robust CR2 standard errors, clustering on the patient; categorical outcomes were compared by Fisher’s exact test with the Freeman–Halton extension. For the two categorical rows the contrast column reports the difference in percentage points. * For absolute ECD, polymegathism and hexagonality the *p* value refers to the MA60AC versus Eyecryl contrast; for every other row it is the omnibus three-group effect. Significant pairwise contrasts. Decentration: MA60AC exceeded Eyecryl after Tukey adjustment. ECD loss at 12 months: Eyecryl versus Sensar *p* = 0.003 and Eyecryl versus MA60AC *p* < 0.001. ECD loss at 24 months: all three contrasts met the Bonferroni-corrected threshold of 0.0167. Visual acuity gain was equivalent within the ±0.15 logMAR margin by two one-sided tests (*p* = 0.009). No other row showed a significant pairwise contrast. BCVA, best-corrected visual acuity; CI, confidence interval; D, dioptres; ECD, endothelial cell density; IOL, intraocular lens; IPTW, inverse probability of treatment weighting; OCT, optical coherence tomography; SD, standard deviation.

**Table 4:** IPTW-weighted multivariable linear regression models predicting 24-month endothelial cell density loss (%).

Predictor	β (%)	Robust SE	95% CI	*p*	Tolerance	VIF
*Model 1: intermediate variables excluded (R* ^2^ *= 0.524; adjusted R* ^2^ *= 0.503; F(6, 138) = 25.3; p < 0.001)*						
AcrySof MA60AC versus Eyecryl	2.140	0.408	1.340 to 2.940	<0.001	0.428	2.34
Sensar AR40e versus Eyecryl	1.180	0.357	0.480 to 1.880	0.001	0.612	1.63
Age, per year	0.004	0.006	−0.008 to 0.016	0.512	0.885	1.13
Axial length, per mm	0.045	0.079	−0.110 to 0.200	0.568	0.912	1.10
Preoperative ECD, per cell/mm^2^	−0.0012	0.0006	−0.0023 to −0.0001	0.039	0.862	1.16
Pars plana vitrectomy versus anterior vitrectomy	−0.412	0.208	−0.820 to −0.004	0.048	0.815	1.23
*Model 2: intermediate variables included (R* ^2^ *= 0.708; adjusted R* ^2^ *= 0.688; F(9, 135) = 36.4; p < 0.001)*						
AcrySof MA60AC versus Eyecryl	0.398	0.266	−0.124 to 0.920	0.136	0.185	5.40 ‡
Sensar AR40e versus Eyecryl	0.534	0.281	−0.017 to 1.085	0.057	0.351	2.85
Operative duration, per minute	0.042	0.0056	0.031 to 0.053	<0.001	0.442	2.26
Anatomical anterior chamber depth, per mm	−1.280	0.183	−1.639 to −0.921	<0.001	0.518	1.93
Euclidean decentration, per mm	0.465	0.187	0.098 to 0.832	0.014	0.694	1.44
Age, per year	0.002	0.005	−0.007 to 0.011	0.681	0.893	1.12
Axial length, per mm	0.021	0.064	−0.105 to 0.147	0.742	0.847	1.18
Preoperative ECD, per cell/mm^2^	−0.0011	0.0005	−0.0021 to −0.0001	0.041	0.806	1.24
Pars plana vitrectomy versus anterior vitrectomy	−0.395	0.117	−0.625 to −0.165	0.001	0.758	1.32

Dependent variable in both models is 24-month percentage ECD loss. Both models are IPTW-weighted with Huber–White robust standard errors. Eyecryl is the reference category for IOL platform. ‡ VIF exceeds the prespecified threshold of 3.0, an expected consequence of entering an exposure indicator alongside variables strongly associated with it; Model 2 coefficients should be interpreted as a set rather than as separately identified contributions. The MA60AC coefficient fell by 81.4% and the Sensar coefficient by 54.7% between the two models. This is descriptive coefficient attenuation, not a causal mediation analysis: no indirect effect was estimated and the intermediate variables were measured contemporaneously with the outcome. CI, confidence interval; ECD, endothelial cell density; IOL, intraocular lens; IPTW, inverse probability of treatment weighting; SE, standard error; VIF, variance inflation factor.

**Table 5:** Intraoperative findings, postoperative complications and attrition.

Category and Event	Eyecryl	Sensar AR40e	AcrySof MA60AC	*p*
*Intraoperative flange creation and haptic integrity (denominator 48/48/49 analysed eyes)*				
More than one cautery application required at either haptic, *n* (%)	2 (4.2)	11 (22.9)	21 (42.9)	<0.001
Haptic fracture or flange failure in a retained eye, *n* (%)	0 (0.0)	1 (2.1)	4 (8.2)	0.127
*Intraoperative conversion (denominator 64/66/66 eyes taken to surgery)*				
Conversion to an alternative fixation strategy, *n* (%)	0 (0.0)	1 (1.5)	3 (4.5)	0.327
*Early postoperative complications within 1 month (denominator 48/48/49 analysed eyes)*				
Corneal oedema persisting beyond 1 week, *n* (%)	3 (6.3)	4 (8.3)	5 (10.2)	0.929
Transient intraocular pressure rise above 25 mmHg, *n* (%)	2 (4.2)	3 (6.3)	3 (6.1)	1.000
Hyphaema or fibrinous anterior chamber reaction, *n* (%)	1 (2.1)	2 (4.2)	2 (4.1)	1.000
*Late postoperative complications beyond 1 month (denominator 48/48/49 analysed eyes)*				
Symptomatic decentration requiring surgical repositioning, *n* (%)	4 (8.3)	6 (12.5)	7 (14.3)	0.726
Flange erosion or exposure, *n* (%)	0 (0.0)	1 (2.1)	1 (2.0)	1.000
Cystoid macular oedema, *n* (%)	1 (2.1)	2 (4.2)	3 (6.1)	0.871
Patients with at least one complication (composite), *n* (%)	9 (18.8)	11 (22.9)	15 (30.6)	0.422
*Serious or permanent adverse events (denominator 48/48/49 analysed eyes)*				
Chronic bullous keratopathy or corneal decompensation, *n* (%)	0 (0.0)	0 (0.0)	0 (0.0)	1.000
Endophthalmitis, *n* (%)	0 (0.0)	0 (0.0)	0 (0.0)	1.000
Complete IOL dislocation, *n* (%)	0 (0.0)	0 (0.0)	0 (0.0)	1.000
*Attrition and reasons for loss to follow-up (denominator 64/65/63 eyes receiving the assigned IOL)*				
Geographic relocation or loss of contact, *n* (%)	8 (12.5)	8 (12.3)	6 (9.5)	0.882
Voluntary withdrawal, *n* (%)	5 (7.8)	5 (7.7)	5 (7.9)	1.000
Development of a new exclusion criterion, *n* (%)	3 (4.7)	3 (4.6)	3 (4.8)	1.000
Death from unrelated causes, *n* (%)	0 (0.0)	1 (1.5)	0 (0.0)	1.000
Total attrition, *n* (%)	16 (25.0)	17 (26.2)	14 (22.2)	0.870

Fisher’s exact test with the Freeman–Halton extension for three groups; total attrition compared by the chi-squared test (chi-squared = 0.28, df = 2). Rows with identical counts in all three groups, and rows with no event in any group, return an exact *p* of 1.000 by construction. Denominators differ between sections and are stated in each section heading. Intraoperative conversions are reported against all eyes taken to surgery because they occurred before an eye could enter the analysed cohort. Because some patients experienced more than one event, individual event counts exceed the number of patients contributing to the composite endpoint. Every intraoperative haptic fracture and every conversion occurred in an eye with a PMMA haptic. IOL, intraocular lens; PMMA, polymethyl methacrylate.

## Data Availability

The de-identified analysis dataset and complete analysis code are deposited in Zenodo (https://doi.org/10.5281/zenodo.22062853). No direct or indirect identifiers are included; dates are expressed as intervals from baseline and the record does not permit re-identification.

## Supplementary Materials

The following supporting information can be downloaded at: https://www.mdpi.com/article/10.3390/medicina62101985/s1, Table S1: STROBE statement—checklist of items that should be included in reports of cohort studies; Table S2: Prespecified exploratory comparison of eyes with preoperative endothelial cell density below 1800 cells/mm^2^ versus 1800 cells/mm^2^ or above; Figure S1: Distribution of stabilised inverse probability of treatment weights; Figure S2: Distribution of preoperative endothelial cell density.
