# Supplementary material for: Comparative Two-Year Outcomes of Three Different Three-Piece Intraocular Lenses in Flanged Intrascleral Fixation
Source: Medicina (Kaunas). 2026 Oct 14;62(10):1985. doi: 10.3390/medicina62101985 (PMC13706697; doi:10.3390/medicina62101985)
Supplement: Supplementary file 1 [file medicina-62-01985-s001.zip › medicina-4549921-supplementary.pdf]

Supplementary Materials

Comparative Two-Year Outcomes of Three Different Three-Piece Intraocular Lenses in Flanged Intrascleral Fixation

Burhan Başkan and Yusuf Evcimen

Table S1. STROBE statement—checklist of items that should be included in reports of cohort studies.

Section/Topic	Item No.	Recommendation	Reported in Manuscript
**Title and abstract**	1	(a) Indicate the study's design with a commonly used term in the title or the abstract	Abstract (Materials and Methods); Section 2.1
		(b) Provide in the abstract an informative and balanced summary of what was done and what was found	Abstract
**INTRODUCTION**			
**Background/rationale**	2	Explain the scientific background and rationale for the investigation being reported	Section 1
**Objectives**	3	State specific objectives, including any prespecified hypotheses	Section 1 (final paragraph)
**METHODS**			
**Study design**	4	Present key elements of study design early in the paper	Section 2.1
**Setting**	5	Describe the setting, locations, and relevant dates, including periods of recruitment, exposure, follow-up, and data collection	Sections 2.1, 2.2, 2.9
**Participants**	6	(a) Give the eligibility criteria, and the sources and methods of selection of participants. Describe methods of follow-up	Sections 2.2, 2.9; Figure 1
		(b) For matched studies, give matching criteria and number of exposed and unexposed	Not applicable (no matching; IPTW, Section 2.3)
**Variables**	7	Clearly define all outcomes, exposures, predictors, potential confounders, and effect modifiers. Give diagnostic criteria, if applicable	Sections 2.3–2.6, 2.8, 2.10–2.14
**Data sources/measurement**	8*	For each variable of interest, give sources of data and details of methods of assessment (measurement). Describe comparability of assessment methods if there is more than one group	Sections 2.5, 2.8, 2.10, 2.11
**Bias**	9	Describe any efforts to address potential sources of bias	Sections 2.3, 2.7, 2.10, 2.11, 2.13
**Study size**	10	Explain how the study size was arrived at	Section 2.13
**Quantitative variables**	11	Explain how quantitative variables were handled in the analyses. If applicable, describe which groupings were chosen and why	Sections 2.5, 2.11–2.14
**Statistical methods**	12	(a) Describe all statistical methods, including those used to control for confounding	Sections 2.13, 2.14
		(b) Describe any methods used to examine subgroups and interactions	Sections 2.8, 2.13; Table S2
		(c) Explain how missing data were addressed	Section 2.13

Section/Topic	Item No.	Recommendation	Reported in Manuscript
		(d) If applicable, explain how loss to follow-up was addressed	Section 2.13
		(e) Describe any sensitivity analyses	Sections 2.13, 3.9
**RESULTS**			
**Participants**	13*	(a) Report numbers of individuals at each stage of study—e.g., numbers potentially eligible, examined for eligibility, confirmed eligible, included in the study, completing follow-up, and analysed	Sections 2.2, 3.1; Figure 1
		(b) Give reasons for non-participation at each stage	Sections 2.2, 3.1; Figure 1; Table 5
		(c) Consider use of a flow diagram	Figure 1
**Descriptive data**	14*	(a) Give characteristics of study participants (e.g., demographic, clinical, social) and information on exposures and potential confounders	Sections 2.3, 3.1; Table 1; Figure S2
		(b) Indicate number of participants with missing data for each variable of interest	Section 3.1; Figure 1 (complete-case cohort; attrition by group in Table 5)
		(c) Summarise follow-up time (e.g., average and total amount)	Sections 2.9, 3.1
**Outcome data**	15*	Report numbers of outcome events or summary measures over time	Sections 3.2–3.9; Tables 2, 3 and 5; Figure 2
**Main results**	16	(a) Give unadjusted estimates and, if applicable, confounder-adjusted estimates and their precision (e.g., 95% confidence interval). Make clear which confounders were adjusted for and why they were included	Sections 2.13, 2.14, 3.2, 3.4, 3.5, 3.8; Tables 3 and 4; Figure S1
		(b) Report category boundaries when continuous variables were categorized	Sections 2.12, 2.13, 3.7; Table S2
		(c) If relevant, consider translating estimates of relative risk into absolute risk for a meaningful time period	Not applicable
**Other analyses**	17	Report other analyses done—e.g., analyses of subgroups and interactions, and sensitivity analyses	Sections 3.3, 3.6–3.9; Table 2; Table S2
**DISCUSSION**			
**Key results**	18	Summarise key results with reference to study objectives	Section 4 (first paragraph)
**Limitations**	19	Discuss limitations of the study, taking into account sources of potential bias or imprecision. Discuss both direction and magnitude of any potential bias	Section 4 (limitations paragraph)
**Interpretation**	20	Give a cautious overall interpretation of results considering objectives, limitations, multiplicity of analyses, results from similar studies, and other relevant evidence	Sections 4, 5
**Generalisability**	21	Discuss the generalisability (external validity) of the study results	Section 4 (limitations paragraph); Section 5
**OTHER INFORMATION**			

Section/Topic	Item No.	Recommendation	Reported in Manuscript
**Funding**	22	Give the source of funding and the role of the funders for the present study and, if applicable, for the original study on which the present article is based	Funding statement

* Give information separately for exposed and unexposed groups. Section numbers refer to the main manuscript. Checklist adapted from von Elm, E.; Altman, D.G.; Egger, M.; Pocock, S.J.; Gøtzsche, P.C.; Vandenbroucke, J.P. The Strengthening the Reporting of Observational Studies in Epidemiology (STROBE) statement: guidelines for reporting observational studies. Lancet 2007, 370, 1453–1457 (www.strobe-statement.org), distributed under the Creative Commons Attribution License.

Table S2. Prespecified exploratory comparison of eyes with preoperative endothelial cell density below 1800 cells/mm² versus 1800 cells/mm² or above.

Parameter	Preoperative ECD <1800 cells/mm² (n = 16)	Preoperative ECD ≥1800 cells/mm² (n = 129)	SMD	p
Study sample and implant allocation				
Eyecryl 3-Piece, n (%)	5 (31.2)	43 (33.3)	—	0.95
Sensar OptiBlue AR40e, n (%)	5 (31.2)	43 (33.3)	—	
AcrySof MA60AC, n (%)	6 (37.5)	43 (33.3)	—	
Baseline characteristics				
Age, years	54.5 ± 16.0	49.7 ± 23.4	0.24	0.42
Male sex, n (%)	10 (62.5)	61 (47.3)	—	0.30
Axial length, mm	22.59 ± 0.94	22.90 ± 1.29	0.27	0.42
Preoperative anterior chamber depth, mm	3.18 ± 0.43	3.12 ± 0.31	0.18	0.98
Preoperative ECD, cells/mm²	1682 ± 90	2104 ± 168	3.14	<0.001
Preoperative BCVA, logMAR	0.50 ± 0.17	0.56 ± 0.20	0.29	0.56
Aphakia (traumatic or iatrogenic), n (%)	9 (56.2)	69 (53.5)	—	0.90
Crystalline lens subluxation, n (%)	3 (18.8)	21 (16.3)	—	
IOL and capsular bag complex dislocation, n (%)	4 (25.0)	39 (30.2)	—	
Pars plana vitrectomy, n (%)	1 (6.2)	28 (21.7)	—	0.20
Intraoperative course				
Operative time, min	17.4 ± 8.2	18.1 ± 7.7	0.09	0.62
More than one cautery application at either haptic, n (%)	5 (31.2)	29 (22.5)	—	0.53
Outcomes at 24 months				
BCVA gain from baseline, logMAR	0.25 ± 0.11	0.31 ± 0.14	0.54	0.07
Absolute BCVA, logMAR	0.26 ± 0.11	0.24 ± 0.13	0.12	0.60
Endothelial cell density loss, % (primary endpoint)	6.48 ± 1.91	6.16 ± 2.10	0.16	0.56
Absolute ECD, cells/mm²	1573 ± 95	1975 ± 168	2.95	<0.001
Euclidean decentration, mm	0.64 ± 0.13	0.66 ± 0.22	0.11	0.73
Anatomical anterior chamber depth, mm	3.49 ± 0.40	3.52 ± 0.37	0.07	0.75
IOL tilt, degrees	3.26 ± 2.22	3.38 ± 2.10	0.06	0.98
Symptomatic decentration requiring surgical repositioning, n (%)	1 (6.2)	16 (12.4)	—	0.69
Exploratory: 24-month percentage ECD loss by implant platform within each stratum				
**preoperative ECD <1800 cells/mm² stratum:**				
Eyecryl 3-Piece	5 eyes	5.76 ± 1.67%		
Sensar OptiBlue AR40e	5 eyes	6.32 ± 1.53%		
AcrySof MA60AC	6 eyes	7.22 ± 2.36%		
Kruskal–Wallis across platforms within the preoperative ECD <1800 cells/mm² stratum: p = 0.30.				
**preoperative ECD ≥1800 cells/mm² stratum:**				
Eyecryl 3-Piece	43 eyes	4.82 ± 1.49%		
Sensar OptiBlue AR40e	43 eyes	6.16 ± 1.92%		
AcrySof MA60AC	43 eyes	7.48 ± 1.99%		
Kruskal–Wallis across platforms within the preoperative ECD ≥1800 cells/mm² stratum: p < 0.001.				

Values are mean ± SD or n (%). Continuous variables were compared by the Mann–Whitney U test and categorical variables by Fisher's exact test; the two multi-category distributions (implant platform, surgical indication) were compared by a chi-squared test and the resulting p value is shown once per block. SMD denotes the standardised mean difference between strata. All values are unweighted. The comparison was prespecified as exploratory and is reported for completeness only: no adjustment for multiplicity was applied, and with 16 eyes in the low-ECD stratum the family-wise type I error rate for the secondary endpoints as a set is substantially above 0.05. The analysis is therefore descriptive and is not used to support any conclusion. Absolute 24-month ECD was derived as preoperative ECD × (1 − fractional 24-month loss). ECD, endothelial cell density; IOL, intraocular lens; SMD, standardised mean difference.

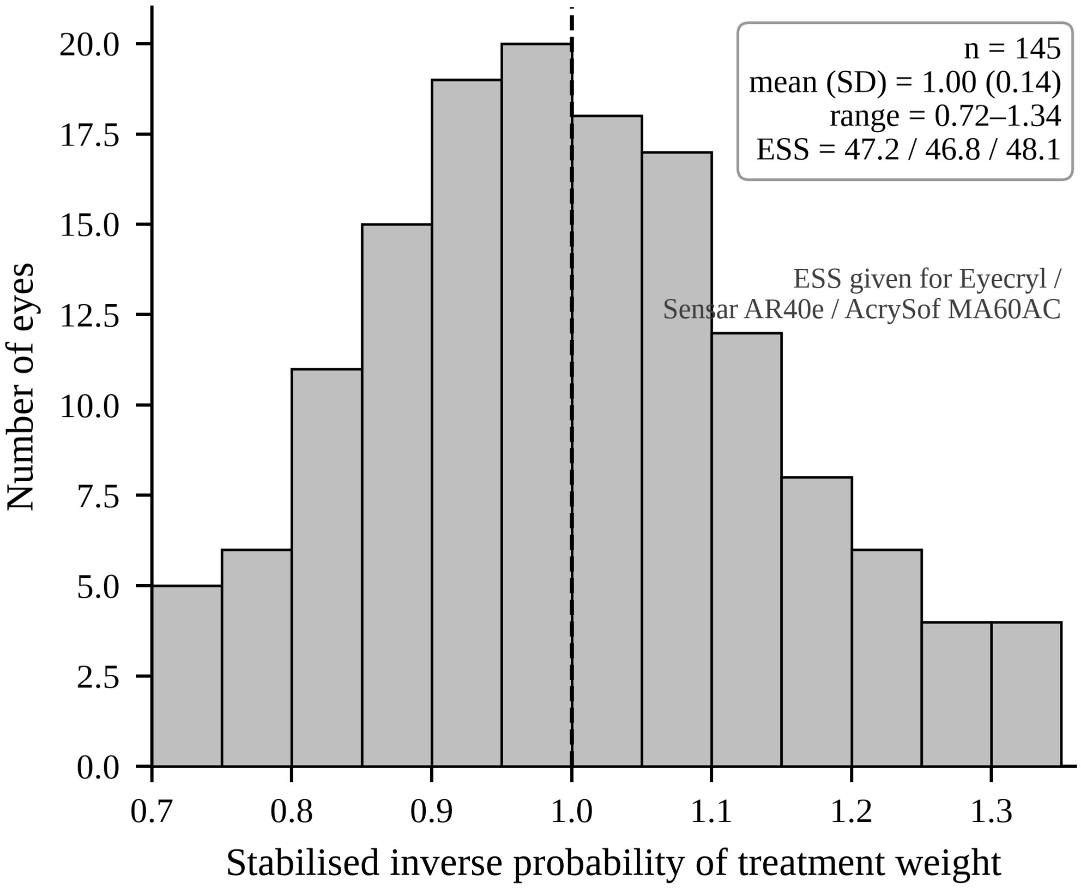

Figure S1. Distribution of stabilised inverse probability of treatment weights in the primary analytical cohort (n = 145). Weights were derived from a generalised propensity score (multinomial logistic regression including age, sex, axial length, preoperative ECD, preoperative BCVA, surgical indication, vitrectomy modality and baseline anterior chamber depth) and truncated at the 1st and 99th percentiles. The dashed line marks a weight of 1.0. Effective sample sizes after weighting were 47.2 (Eyecryl), 46.8 (Sensar AR40e) and 48.1 (AcrySof MA60AC). BCVA, best-corrected visual acuity; ECD, endothelial cell density; ESS, effective sample size; SD, standard deviation.

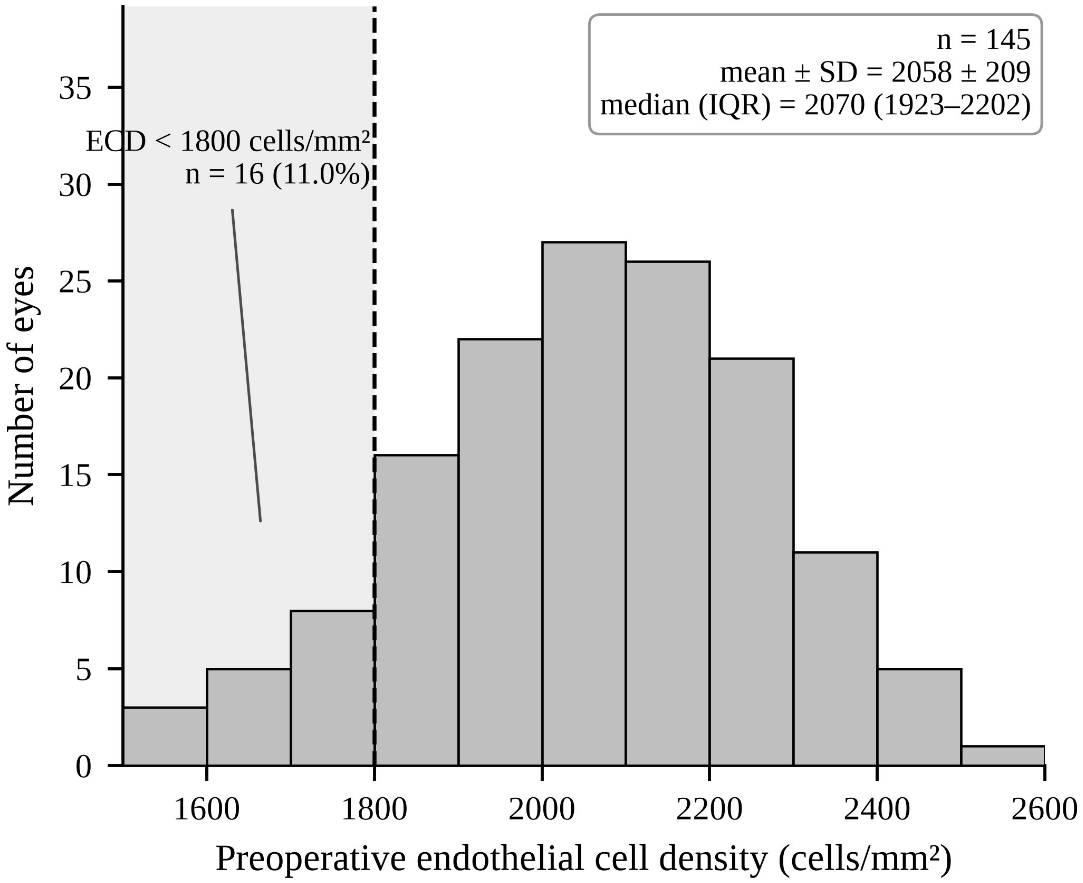

Figure S2. Distribution of preoperative endothelial cell density in the primary analytical cohort (n = 145). The dashed line marks the prespecified threshold of 1800 cells/mm²; the shaded region contains the 16 eyes (11.0%) below this threshold that form the low-ECD stratum in Table S2. ECD, endothelial cell density; IQR, interquartile range; SD, standard deviation.
